# Multisensory nature-based Recharge Rooms’ effect on healthcare workers in a multicenter study

**DOI:** 10.3389/fpsyg.2025.1706772

**Published:** 2026-01-05

**Authors:** Lily Cooke, Arianna Fiorentino, Abbey Sawyer, Neha S. Dangayach, Scott Sharples, Rena Szabo, B. Wade Hamilton, Mar Cortes, David Putrino

**Affiliations:** 1 Department of Rehabilitation and Human Performance, Icahn School of Medicine at Mount Sinai, New York, NY, United States; 2 Department of Neurosurgery, Icahn School of Medicine at Mount Sinai, New York, NY, United States; 3 Banner Health, Phoenix, AZ, United States; 4 Vanderbilt University School of Medicine, Nashville, TN, United States; 5 Hospital Universitario Donostia, San Sebastián, Spain

**Keywords:** COVID-19, stress, burnout, trauma, biophilic design, healthcare

## Abstract

**Background:**

Healthcare workers frequently experience significant levels of stress impacting wellbeing and performance. A previous single site study showed multisensory, naturalistic “Recharge Rooms” were associated with a self-reported improvement in stress across healthcare workers. Subsequently, rooms were constructed at multiple hospitals across the country to assess their effects in a multi-site study.

**Objectives:**

To investigate the association between the Recharge Rooms and self-reported stress levels, alertness, and mood of healthcare workers in multiple health centers.

**Methods:**

Underutilized spaces across 18 US hospitals were converted into Recharge Rooms using video projections of nature landscapes, silk imitation plants, essential oil diffusers, low lighting, music, and nature sounds to create an immersive atmosphere. Healthcare staff had 24/7 access to the rooms. Stress, hopefulness, and alertness were assessed pre- and post-experience on a 0–100 scale with 100 representing “extreme” and 0 being “not at all.”

**Results:**

Of 739 healthcare workers who scheduled time in the rooms, 563 (76%) completed the survey. Average self-reported stress scores decreased from 63.2 to 25.8 (59.1% reduction, *p* < 0.0001). In addition, the average self-reported hopefulness and alertness increased by 29.6% and 35.1% after a session, respectively (*p* < 0.0001).

**Discussion:**

The Recharge Rooms were well-received and associated with immediate, self-reported reductions in perceived stress. The self-reported improvement in perceived stress, mood, and alertness were promising results of this multisite, survey-based evaluation of the technology. Controlled trials using validated instruments are necessary to confirm these findings, assess long-term outcomes, and to better understand the physiological effects of this employee wellness intervention.

## 1 Introduction

Attention Restoration Theory (ART) proposes that natural environments can be restorative, with exposure to nature helping to reduce cognitive fatigue and the ability to concentrate (Kaplan, 1995). Experiences in nature allow the brain to rest and replenish executive functioning by moving processing to the instinctive reptilian brain. This reduces performance pressure, which is especially important for physicians and other professionals whose roles involve prolonged periods of directed attention. Bio-experiential design builds on ART through the design and development of environments that incorporate nature-based elements, supporting cognitive performance, such as attention control and concentration (Bowler et al., 2010; Berto, 2014; Lymeus et al., 2018). Beneficial applications of such designs have been previously demonstrated across stress/trauma, anxiety and depression, dementia, stroke, and other brain injury (Ulrich et al., 1991; Berman et al., 2012; Wilker et al., 2014; Vibholm et al., 2020; Peters and Verderber, 2022). Furthermore, exposure to natural features have been shown to improve heart rate, blood pressure, mental health, the immune system, the microbiome, and asthma symptoms, while also reducing mortality (Hartig et al., 2003; Laumann et al., 2003; Mitchell and Popham, 2008; Li, 2010; Wilker et al., 2014; Sobko et al., 2020; Cavaleiro Rufo et al., 2021).

Our environments can have a significant impact on our emotional and mental wellbeing. Studies show that urban environments with loud noises, bland architecture, and lack of green space negatively impact mental health (Hedblom et al., 2017, 2019). Conversely, spaces incorporating biophilic or bio-experiential design can positively influence psychological wellbeing (Bolten and Barbiero, 2020). Elements of bio-experiential design are currently being applied in many settings including schools, office buildings, and, importantly, hospitals (Gray and Birrell, 2014; Aristizabal et al., 2021; You et al., 2023; Guidolin et al., 2024; Seale et al., 2024). Traditional hospital settings are viewed as cold and sterile, but decades of research prove such spaces are not conducive to healing (Park and Mattson, 2009; Pati et al., 2016). Simply changing the perception of a patient waiting room from a hospital-like setting to a healing space incorporating biophilic principles can decrease anxiety and emotional distress (Baldwin, 2012; DuBose et al., 2018).

Everyone who shares space in a hospital setting stands to benefit from spaces intentionally designed to promote their wellbeing. Clinician burnout, characterized by reduced professional efficacy, depersonalization, and emotional exhaustion, has been increasing in the United States long before the COVID-19 pandemic arrived (Maslach et al., 2001; Amanullah and Ramesh Shankar, 2020). The pandemic exacerbated this crisis, as frontline workers faced resource shortages, extreme workloads, all while navigating severe disruptions to daily life outside of work (Morgantini et al., 2020). Taken together, these factors compound negative emotions and moral injury that can contribute to burnout in frontline healthcare workers, and clinician burnout numbers have largely increased since the COVID-19 pandemic (Linzer et al., 2022).

Given increasing rates of burnout, and the potential consequences for patient safety and care quality, there is an urgent, current need for simple, scalable interventions that reduce stress in healthcare workers (Hall et al., 2016; Jun et al., 2021). Physical environments that are designed to passively reduce stress and improve mood may be advantageous to other hospital wellness initiatives because they have no entry-level or training requirements to convey benefits. During the first COVID-19 surge of 2020, our team developed spaces called “Recharge Rooms” in a New York City hospital to provide stress relief to overwhelmed healthcare workers. These immersive, private spaces were intentionally designed to address trauma, anxiety, and stress based on biophilic design principles and resulted in sizable and significant reductions in self-reported stress levels in healthcare workers (Putrino et al., 2020). Building on this initial pilot, the current study expands the evaluation of the Recharge Rooms across 18 hospitals in the United States. The goal of the present work was to use survey instruments to evaluate the benefits experienced by Recharge Room users across the country and explore the extent to which the benefits observed in a single-site investigation are maintained at scale. We hypothesize that the Recharge Rooms will improve stress, mood, and alertness in this multicenter study.

## 2 Materials and methods

The present work conducts analysis of anonymous survey data collected for the purposes of quality improvement of wellness initiatives offered to hospital workers across the country. Surveys were entirely voluntary and were not required to be completed for access to the Recharge Rooms. As the work was a quality improvement initiative, it was exempt from ethical review and approval.

### 2.1 Participants

Healthcare workers of all locations were invited to participate in the Recharge Room experiences including medical doctors, nurses, researchers, maintenance workers, security, volunteers, students, and culinary team.

### 2.2 Recharge Rooms

The Recharge Rooms were set up in 18 different sites across the country in six different states (Arizona, Baltimore, Colorado, Michigan, New York, Virginia) to relieve healthcare worker stress and trauma. The rooms were constructed based on ART principles, incorporating scenes of nature and other sensory experiences to create an atmosphere that provides physiological and psychological benefits.

All of the materials used to build the rooms were sourced according to healthcare environment standards. Other items, including but not limited to projectors, imitation plants, and essential oil diffusers were carefully curated and sourced based on their durability in a 24/7 environment. Using a voice activated edge computing device that integrates with immersive audio visual devices, including a projector, speakers and lighting (Soluna^®^, Studio Elsewhere, Brooklyn, NY, United States), healthcare workers were able to change the nature scenery on a blank wall without having to interact with screens or touch any items in the room, minimizing user interaction with surfaces. The Hue Bridge (Phillips, Netherlands) lighting system was programmed to synchronize with the different projected nature scenes (i.e., Hue lights would turn blue for ocean scenes and green for forest scenes). High-definition audio recordings of nature sounds were also paired with appropriate music.

Additionally, an essential oil diffuser or individual essential oil inhalers were used to create scent profiles associated with the depicted nature landscape. These particular essential oils were chosen due to their positive impact on stress relief and their soothing effects (Matsumoto et al., 2014; Ali et al., 2015; Ikei et al., 2015). Silk imitation plants created the impression of a green space and were arranged in a semi-circular pattern behind the available seating. This was done to create the impression of being fully surrounded by and immersed in a natural environment. All materials were non-porous and could be quickly sanitized after each use for infection control purposes. The above elements were chosen due to their reported benefits on anxiety and stress (Kellert, 2008).

Information about the Recharge Rooms, including a description of the overall environment, and the hours of operation, was distributed to staff.

### 2.3 Outcome measures and study design

Before entering the Recharge Rooms for their scheduled appointment, users were prompted to complete an author-developed survey of their stress level, mood change, and cognitive alertness. Users were asked to only complete the survey a single time even if they were frequent Recharge Room users. These self-reported measures were based on a 0–100 scale with 0 being “calm or not stressed at all” and 100 being “extremely stressed.” Mood change was measured from hopeless to hopeful and cognitive alertness ranged from drowsy to alert on the 0–100 scale.

Upon completion of a 15-min experience in the Recharge Rooms, when exiting users were again prompted to complete the outcome measures. The participants also answered statements relating to their experience in the Recharge Room (Questions 1–5, Table 1), in addition to questions on overall satisfaction (Question 6, Table 1) and a measure of user experience using the Net Promoter Score (NPS) (Question 7, Table 1), which were all scored from 0 to 100 (Reichheld, 2003). Although NPS is most useful when placed in the context of NPS scores of competing programs, the creators of the scale consider scores above 20 as “favorable,” scores above 50 as “excellent,” and scores above 80 as “world class” (Net Promoter Score, 2025). Furthermore, they were asked to provide more details about their visits to the Recharge Room, such as with whom they experienced the room, how many times, and for how long (Table 1). Finally, respondents were given the option of providing additional comments in an open-ended “additional comments” section.

**TABLE 1:** Post-room experience and visit characteristics questionnaire.

Post-room experience questionnaire		
	Questions	Responses
1.	I was able to mentally process challenging emotions	(0–100 scale)
2.	I sensed things momentarily slow down	(0–100 scale)
3.	I felt connected to humanity	(0–100 scale)
4.	I felt a sense of togetherness with my colleagues	(0–100 scale)
5.	I had goosebumps, chills, or shivers up my spine	(0–100 scale)
6.	How satisfied are you with your overall experience?	(0–100 scale)
7.	How likely are you to recommend this to a colleague?	(0–100 scale)
**Visit characteristics questionnaire**		
1.	How many times have you experienced the room?	Up to 5 times 6–10 times More than 10 times
2.	How long was your session?	<10 min 10–20 min >20 min
3.	How did you experience the room?	Alone With coworkers Went alone, others were present

### 2.4 Statistical analysis

Survey data was gathered from all users of the rooms from October 29th, 2021 to February 1st, 2022. Descriptive statistics and the NPS were calculated, alongside a paired *t-*test to quantify changes in stress levels, mood, and cognitive alertness. All the analyses were performed using Excel (2016 version) and SPSS software (IBM SPSS Statistics for Windows, Version 31.0. Armonk, NY: IBM Corp). Since some responses are missing or unreported, the total count of the responses is provided where applicable.

## 3 Results

### 3.1 Participation

Among the 739 healthcare workers who scheduled time to visit the space in the 3-month survey period across the eighteen centers, 563 (76%) completed the survey.

### 3.2 Wellbeing outcomes

**FIGURE 1:**
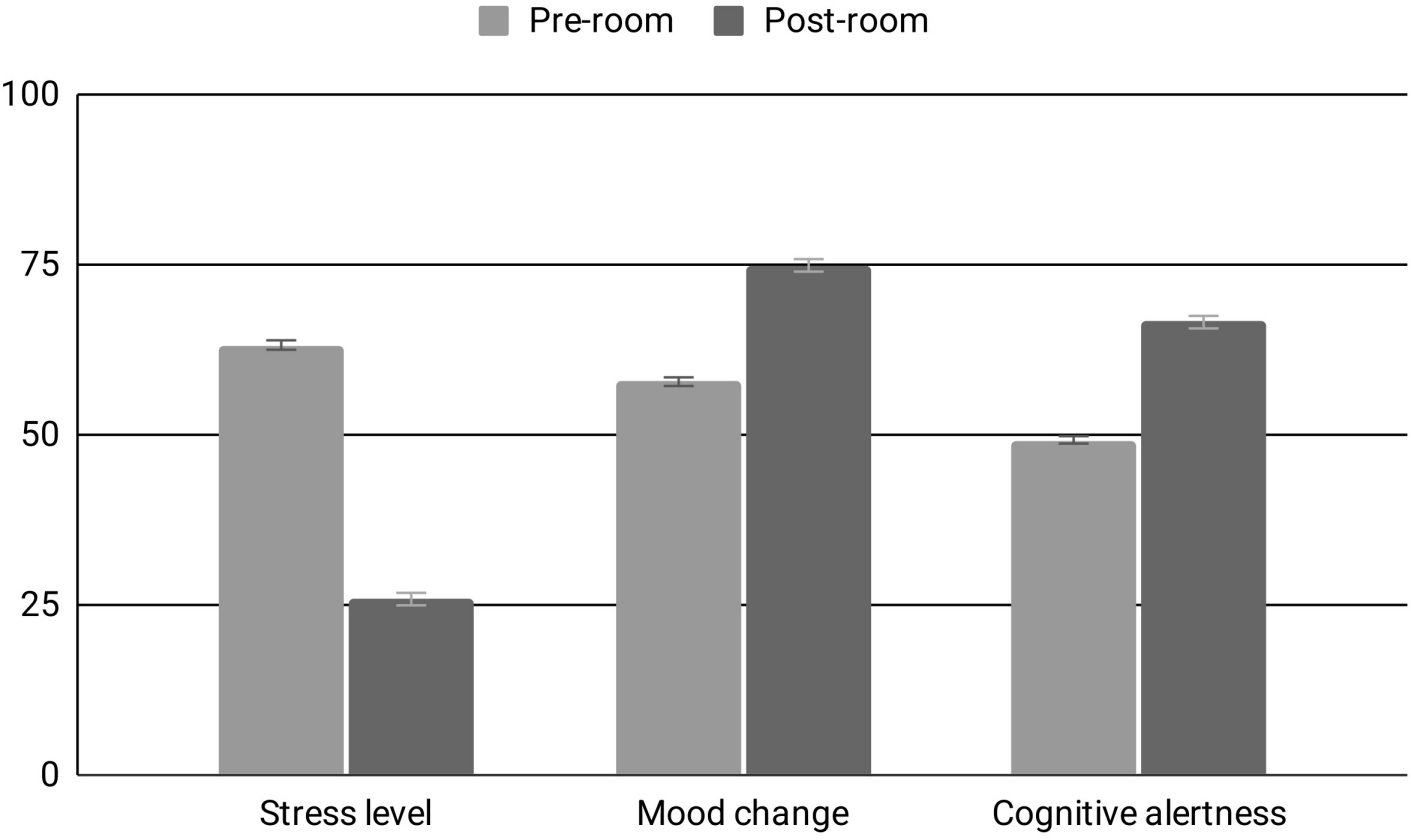
Well-being outcomes, pre- and post-room exposure. Analyzed sample: Stress level (pre, *n* = 558; post, *n* = 557), Mood change (pre, *n* = 549; post, *n* = 556), Cognitive alertness (pre, *n* = 557; post, *n* = 556). Asterisks indicate significant within-group change.

Regarding the three main outcomes, participants self-reported a 59.1% decrease in stress on the calm-stressed scale (63.17 ± 1.17 pre-experience, 25.84 ± 0.92 post-experience; t(551) = 31.38, *p* < 0.0001, *d* = 1.33, 95% CI, 39.31–34.68) and a 29.6% increase in the hopefulness measure (57.78 ± 1.10 pre-experience, 74.90 ± 0.91 post-experience; t(543) = 17.84, *p* < 0.0001, *d* = 0.76, 95% CI, 18.82–15.09). For the drowsy-alert scale, participants reported a 35.1% increase in alertness (49.23 ± 1.14 pre-experience, 66.5 ± 1.04 post-experience; t(549) = 15.23, *p* < 0.0001, *d* = 0.65, 95% CI, 19.93–15.38) (Figure 1).

Healthcare workers were given several statements to consider related to their experience in the Recharge Room. The level of agreement with each statement is presented in Table 2.

**TABLE 2:** Extent of agreement with the descriptive statements of participants’ room experience.

Statements	Analyzed sample	Median score	Quintile	Agreement level
I felt connected to humanity	*n* = 552	66	3	Moderate
I felt a sense of togetherness with my colleagues	*n* = 341	64	3	Moderate
I sensed things momentarily slowed down	*n* = 558	77	3	Moderate
I was able to mentally process challenging emotions	*n* = 550	67	3	Moderate
I had goosebumps, chills, or shivers up my spine	*n* = 204	50	3	Moderate

*n*, sample size.

### 3.3 Visit characteristics

Use statistics related to the recharge rooms are detailed in Table 3.

**TABLE 3:** Recharge Room visits characteristics of the analyzed sample.

Variables	Analyzed sample
How many times have you experienced the room?	*n* = 304
Up to 5 times	289 (95.1%)
6–10 times	12 (3.9%)
More than 10 times	3 (1.0%)
How long was your session?	*n* = 535
Less than 10 min	263 (49.2%)
10–20 min	210 (39.3%)
More than 20 min	62 (11.6%)
How did you experience the room?	*n* = 545
Alone	235 (43.1%)
With co-workers	275 (50.5%)
Went alone, others were present	35 (6.4%)

All values are count (percentage). *n*, sample size.

The NPS for the experience was 52, with 61.74% of respondents (*n* = 562) identifying as “promoters” (scores ranging between 9 and 10) of the experience.

### 3.4 Qualitative feedback

Respondents submitted qualitative comments that were overall positive, such as “*Best 20 min of my day!”* or “*Wonderful experience to help calm my mind and get rid of negative thoughts after a rough procedure. I will definitely be recommending this to coworkers who need to recharge or relax.*” Additionally, several comments suggested that users viewed the experience as a gesture of institutional support, e.g., “*So thoughtful to have this at the facility and care about the mental wellbeing of staff*.”

## 4 Discussion

The COVID-19 pandemic highlighted the magnitude of pressures that can be placed upon the minimal capacity buffer available to frontline clinical services, and the severe mental health consequences that can result from these demands (Spoorthy et al., 2020). Results from this multicenter study build on our initial single study design, and show that healthcare workers feel more calm, hopeful, and alert after a Recharge Room experience (Putrino et al., 2020). The high NPS and positive qualitative feedback further supports the feasibility and acceptability of scaling this intervention across hospital systems.

Our findings that the Recharge Rooms significantly improve mood, stress levels, and alertness, provide an effective and feasible route to reducing the clinical burnout that arises from busy, high stress environments. A key feature of the benefit provided by Recharge Room environments is that it is a passive process and requires minimal effort from the user other than to rest quietly and experience the room. By contrast, active processes such as meditation and talk therapy demand familiarity with the process associated with these modalities and continuous process updating and improvement to remain effective (Champagne-Langabeer et al., 2025). Additionally, adherence to at-home meditation and in person talk therapy often varies across lived experience, personality traits, and patient-clinician relationship (Thompson and McCabe, 2012; University of British Columbia and Kambolis, 2017). As such, the passive experience of the Recharge Rooms offers accessible mental health services with a low barrier to entry for healthcare employees, as benefits can be seen with no specific training or regular practice from participants. It should be noted that the Recharge Room is a location-based passive experience, while more active processes such as meditation and talk therapy can be performed anywhere. Taken together, experiences such as the Recharge Rooms could be viewed as a valuable entry-level wellness intervention that does not require much “buy in” from busy healthcare workers, but still provides significant benefit. After experiencing the benefits of Recharge Rooms, users may feel more motivated and willing to invest time in active processes, which have previously experienced issues with uptake and retention in corporate wellness programs (Liu et al., 2025).

The influence of perceived institutional support may have been a potential mechanism in the improved mood and stress outcomes. Institutional support has been shown to improve self-assessed stress, and depressive and traumatic symptoms in healthcare workers during the COVID-19 pandemic (Chatzittofis et al., 2021). Additionally, attentional restoration may have played a role in the improved alertness results. Nature environments have been shown to improve attention control (Bowler et al., 2010; Berto, 2014; Lymeus et al., 2018).

Despite these positive findings, the current program lacks the use of validated outcome measures and a control group, as well as the rigor of a structured clinical trial. The author-developed questionnaires, while informative for initial findings and safe scaling of the program, limit generalizability and rigor, and allows for potential response bias. A further limitation is the profession of service users and other demographics were not collected. Future research should assess the longer-term effects the Recharge Room experience has on the mental health of healthcare workers and other high-risk users, while also implementing validated questionnaires and assessing additional psychological indicators of burnout.

The scalability of the rooms makes them a promising candidate to help address the dearth of effective healthcare worker stress interventions, should these results be replicated in more rigorous study designs (Marine et al., 2006). Moreover, the Recharge Rooms may aid in addressing the lack of passive interventions to improve healthcare worker stress and burnout.

## Data Availability

The raw data supporting the conclusions of this article will be made available by the authors, without undue reservation.
